# Cloud computing for comparative genomics

**DOI:** 10.1186/1471-2105-11-259

**Published:** 2010-05-18

**Authors:** Dennis P Wall, Parul Kudtarkar, Vincent A Fusaro, Rimma Pivovarov, Prasad Patil, Peter J Tonellato

**Affiliations:** 1 Center for Biomedical Informatics, Harvard Medical School, Boston, MA 02115 USA; 2 Department of Pediatrics, Harvard Medical School, Boston, MA 02115, USA

## Abstract

**Background:**

Large comparative genomics studies and tools are becoming increasingly more compute-expensive as the number of available genome sequences continues to rise. The capacity and cost of local computing infrastructures are likely to become prohibitive with the increase, especially as the breadth of questions continues to rise. Alternative computing architectures, in particular cloud computing environments, may help alleviate this increasing pressure and enable fast, large-scale, and cost-effective comparative genomics strategies going forward. To test this, we redesigned a typical comparative genomics algorithm, the reciprocal smallest distance algorithm (RSD), to run within Amazon's Elastic Computing Cloud (EC2). We then employed the RSD-cloud for ortholog calculations across a wide selection of fully sequenced genomes.

**Results:**

We ran more than 300,000 RSD-cloud processes within the EC2. These jobs were farmed simultaneously to 100 high capacity compute nodes using the Amazon Web Service Elastic Map Reduce and included a wide mix of large and small genomes. The total computation time took just under 70 hours and cost a total of $6,302 USD.

**Conclusions:**

The effort to transform existing comparative genomics algorithms from local compute infrastructures is not trivial. However, the speed and flexibility of cloud computing environments provides a substantial boost with manageable cost. The procedure designed to transform the RSD algorithm into a cloud-ready application is readily adaptable to similar comparative genomics problems.

## Background

The onslaught of new genome sequences has begun to outpace the local computing infrastructures used to calculate and store comparative genomic information. For example, because the number of genomes has increased approximately 12 fold over the last 5 years, algorithms that detect orthologs and assemble phylogenetic profiles are faced with an increasing computational demand.

**Figure 1:**
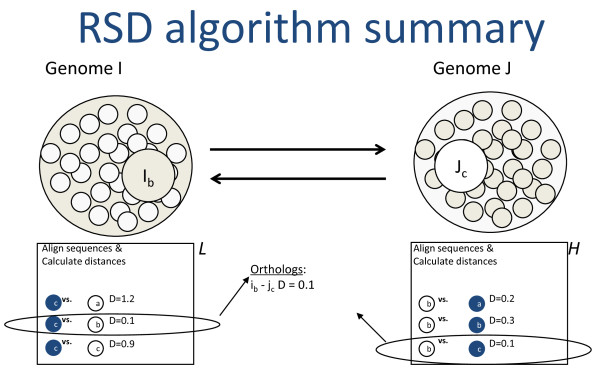
**The reciprocal smallest distance algorithm (RSD)**. Arrows denote bidirectional BLAST runs. After each run, hits are paired with the query to calculate evolutionary distances. If the same pair produces the smallest distance in both search directions, it is assumed to be orthologous. The specifics of the algorithm are provided in the Introduction.

One such computationally intensive comparative genomics method, the reciprocal smallest distance algorithm (RSD), is particularly representative of the scaling problems faced by comparative genomics applications. RSD is a whole-genomic comparative tool designed to detect orthologous sequences between pairs of genomes. The algorithm [1] (Figure 1) employs BLAST [2] as a first step, starting with a subject genome, *J*, and a protein query sequence, *i*, belonging to genome *I*. A set of hits, *H*, exceeding a predefined significance threshold (e.g., E < 10^-10^, though this is adjustable) is obtained. Then, using clustalW [3], each protein sequence in *H *is aligned separately with the original query sequence *i*. If the alignable region of the two sequences exceeds a threshold fraction of the alignment's total length (e.g., 0.8, although this is also adjustable), the codeml program of PAML [4] is used to obtain a maximum likelihood estimate of the number of amino acid substitutions separating the two protein sequences, given an empirical amino acid substitution rate matrix [5]. The model under which a maximum likelihood estimate is obtained in RSD may include variation in evolutionary rate among protein sites, by assuming a gamma distribution of rate across sites and setting the shape parameter of this distribution, α, to a level appropriate for the phylogenetic distance of the species being compared [6]. Of all sequences in *H *for which an evolutionary distance is estimated, only *j*, the sequence yielding the shortest distance, is retained. This sequence *j *is then used for a reciprocal BLAST against genome *I*, retrieving a set of high scoring hits, *L*. If any hit from *L *is the original query sequence, *i*, the distance between *i *and *j *is retrieved from the set of smallest distances calculated previously. The remaining hits from *L *are then separately aligned with *j *and maximum likelihood distance estimates are calculated for these pairs as described above. If the protein sequence from *L *producing the shortest distance to *j *is the original query sequence, *i*, it is assumed that a true orthologous pair has been found and their evolutionary distance is retained (Figure 1).

The algorithm is a multi-step process that composes several applications (Figure 1) into a straightforward workflow. The workflow involves the use of BLAST for initial sequence comparison, clustalW for sequence alignment, codeml for estimation of distance calculation, as well as various intervening conversion programs to ensure proper formatting of input Keeping the tunable parameters of the algorithm constant, RSD scales quadratically with the number of genome sequences. However, to enable more flexibility for ortholog detection among distantly related organisms and also to enable the creation of clusters of recent paralogs, RSD should ideally be run over a range of values for both the divergence and evalue parameters, spanning from conservative to relaxed. Thus, the total number of processes that must be run for N is ((N)(N-1)/2)*M, where M represents the number of different parameter settings for evalue and divergence.

Assuming that the current number of genomes sequences, N, is 1000, and the number of different parameter settings, M, is 12, the total number of processes required for a full complement of results would be 5,994,000. Further assuming that each individual process takes on average 4 hours (generally a lower bound for big genomes), and constant access to 300 cores of computer processing power, the total time to complete this task would be 79,920 hours, or 9.1 years. Therefore, the cost of operation of the RSD algorithm can be quite extensive and magnified not only by the influx of new genome sequences, especially as sequencing technologies continue to improve in speed, efficiency and price, but also by the rate at which genomic sequences are updated and revised. In fact, to keep pace with genome additions and revisions, ensuring all-versus-all for both new and existing genomes, the number of processes rises as: f(N,0) = ((N × 0) + (N × (N-l)/2)) × M, where N is the number of genomes awaiting computation of orthologs, 0 is number of genomes previously processed, and M is the number of different parameter settings.

Elastic cloud architectures, for example Amazon's Elastic Computing Cloud (EC2) [7], represent a possible solution for rapid, real-time delivery of cross-genomic data as the availability of genomic information continues to climb at a rapid pace. Typical uses of the cloud have been in the areas of economics, health, and the entertainment industry, but so far this computing solution has had limited impact on the field of comparative genomics. Only a handful of projects have been launched, for example, the Sanger Institutes fast matching and alignment algorithm to assemble full human genome [8], Cloud Burst to map next generation sequencing data [9], Cloud Blast a "clouded" implementation of NCBI BLAST [10], a virtual laboratory for protein sequence analysis on cloud established at Indiana University [10], and an algorithm to search for single nucleotide polymorphisms [11]. Yet the number of cloud resources is on the rise, with service-based cloud environments from Microsoft [12], Google [13], Amazon [7], SGI [14], and more, lending an unprecedented opportunity to evaluate the capabilities of the cloud for sustainable and large-scale comparative genomics.

In the present study, we elected to test the capabilities of EC2 for all-against-all ortholog calculation using the reciprocal smallest distance algorithm across a wide array of recently sequenced genomes. Our study examines the efficacy of the cloud in general, and the best practices for optimal setup and operation within the EC2 in particular. We expect that the protocols developed as a consequence of our research will be readily scalable to other problems within the space of comparative genomics as well as to other fields employing similar workflows for large-scale computation.

## Results

### Cloud Testing and configuration

Prior to the successful operation of the cloud, it was necessary to choose optimal settings for various parameters used by the Elastic MapReduce framework (EMR), the framework that enables parallel processing within the Elastic Compute Cloud (EC2). The complete configuration of the cloud for both the BLAST and ortholog estimation steps required that 12 parameters be set (summarized in Table 1). The argument "--jobconf mapred.map.tasks" was used to specify *a priori *the number of map tasks for both the blast step and ortholog computation step of the RSD cloud algorithm. In our case, the number of map tasks was the number of BLAST comparisons and number of ortholog computations, respectively. In cases similar to ours, for example, situations where a user is only running BLAST or clustalw, this setting would still need to be used, but adjusted appropriately to equal the number of blast comparisons or clustal alignments required. Since our process flows did not need a reducer step, the output of the mapper task was the final output of each job, and the number of output files (called "part" files in HDFS) generated was equivalent to the total number of mapper tasks.

**Table 1:** Elastic Map Reduce commands

Argument	Description	Input
--stream	Activates the "streaming" module	N/A

--input	File(s) to be processed by EMR	hdfs:///home/hadoop/blast_runner hdfs:///home/hadoop/ortho_runner

--mapper	Name of mapper file	s3n://rsd_bucket/blast_mapper.py s3n://rsd_bucket/ortho_mapper.py

--reducer	None required, reduction done within RSD algorithm	N/A

--cache-archive	Individual symlinks to the executables, genomes,	s3n://rsd_bucket/executables.tar.gz #executables,#genomes, #RSD_standalone,#blastinput,#results

--output		hdfs:///home/hadoop/outl

-- jobconf mapred.map.tasks	Number of blast and ortholog calculation processes	= N

-- jobconf mapred.tasktracker.map.tasks.maximum	Total number of task trackers	= 8

--jobconf mapred. task, timeout	Time at which a process was considered a failure and restarted	= 86400000 ms

--jobconf mapred.tasktracker.expiry.interval	Time at which an instance was declared dead.	3600000 (set to be large to avoid instance shut down with long running jobs)

--jobconf mapred.map.tasks.speculative.execution	If true, EMR will speculate that a job is running slow and run the same job in parallel	False (because the time for each genome-vs-genome run varied widely, we elected to set this argument to False to ensure maximal availability of the cluster)

Specific commands passed through the Ruby command line client to the Elastic MapReduce program (EMR) from Amazon Web Services. The inputs specified correspond to (1) the BLAST step and (2) the ortholog computation step of the RSD cloud algorithm. These configurations settings correspond to both the EMR and Hadoop frameworks, with two exceptions: In EMR, a --j parameter can be used to provide an identifier for the entire cluster, useful only in cases where more than one cloud cluster is needed simultaneously. In Hadoop, these commands are passed directly to the streaming.jar program, obviating the need for the --stream argument.

Certain parameters including "--jobconf mapred.task.timeout" required tests to identify the best value for optimal performance and cost effectiveness of the compute cloud. This parameter was used to specify the maximum number of hours needed to execute the RSD cloud algorithm on a single pair of genomes. If the value for this parameter was set to be too low, ortholog comparisons exceeding this setting were marked as failed by the EMR program causing after 4 consecutive tries the node to be blacklisted by EMR and no longer available for further computational processes. On the other hand, if the value for this parameter was set to be too high, jobs that had failed due to transient filesystem errors or other reasons were left running in a zombie state, thereby burning time and resources. In either case, the size of the compute cloud and the speed of the calculations were negatively impacted. Therefore, we empirically determined an optimal setting for this parameter by benchmarking the time period needed to complete the largest pairs of genomes available in our Roundup data repository [15]. We determined the best "goldilocks" setting to be 86400 seconds (~24 hours). This ensured that the EMR process would distinguish between long-running and failed jobs without impacting the availability of nodes within the cluster.

In addition, the allocation of the heap space was of critical importance to ensure proper function of the compute cloud. Through various test runs we discovered that the JobTracker daemon would frequently run out of memory and require manual restarts. Because this occurred on the master node, the entire cluster would be negatively impacted. To avoid this, we used a bash script that would automatically reconfigure the memory allotted to the daemon at launch time. We placed the script on S3 and passed it to the EMR program using the "--info" option. The script accepted a simple argument designed to reallocate the memory assigned to the JobTracker daemon from the default setting of 1GB to 3GB for BLAST processes and 5GB for RSD processes. These values represented upper bounds and successfully avoided memory-related compute cloud failures.

### Compute cloud processing

We selected 55 small bacterial genomes that had not already been incorporated into the existing Roundup repository [15]. To provide a comprehensive test of the capabilities of the EC2, we computed orthologs for all pairs of these 55 new genomes, plus the number of processes needed to compare these 55 with the existing set of genomes included in the Roundup repository, 399 at the time of writing, bringing the total number of genomes compared to 454. As such, the total number of computational jobs run on the cloud was 328,020 computed as ((N*N-l/2)+(N*399))*2 +CCN*N-l/2)+(N*399))*X, where N is the number of new genomes and X represents the number of parameter combinations typically calculated by the Roundup tool, in this case 12. The first part of the formula corresponds to the BLAST procedure and the second corresponds to the ortholog estimation step. Although the 55 new genomes used for our study were relatively small, the genomes contained in the Roundup repository against which these were compared spanned a wide range of large eukaryotic and smaller bacterial genomes. The smallest genome contained 477 sequences and the largest contained 46892, and the time for completion of any genome comparison ranged from approximately 5 minutes to 4 hours. Table 2 provides a detailed summary of the process time and cost per step.

**Table 2:** Summary of time and cost for Elastic MapReduce runs.

Process Type	processes	instances	Time	Total ($)
Blast	21945	100	40 hours 0 mins	$ 3,680

Ortholog Estimation	281160	100	28 hours 21 mins	$ 2,622

These cost estimates are based on the use of the High-CPU Extra Large Instance at 0.80 per hour and use of EMR at 0.12 per hour. These costs assume constant processing without node failures. Total costs = $6302.

**Figure 2:**
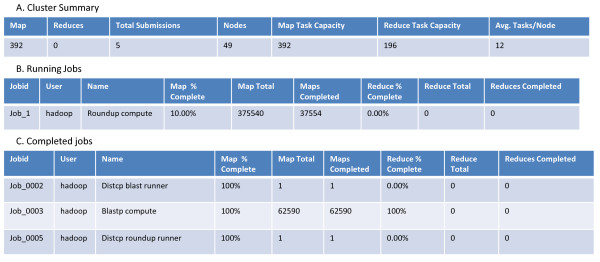
**Example of the Compute Cloud user interface for monitoring the health of the cluster and progress of mapped cloud tasks**. (A) The Cluster summary provided a summary of the compute cloud. (B) Running jobs listed the Job id of the current running task, root user, job name and map task progress update. (C) Completed Jobs provided an up-to-date summary of completed tasks. This user interface also provided information about failed steps as well as links to individual job logs and histories. Access to this user interface was through FoxyProxy, described in the Methods.

**Figure 3:**
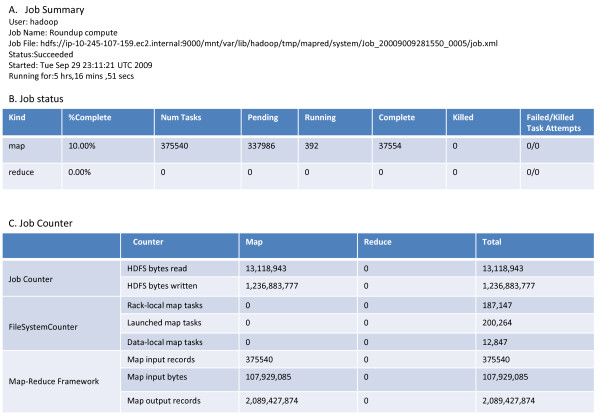
**Example of the Job user interface for monitoring the status of individual jobs**. (A) Job summary provided job information like the user, job start time and the duration of the job. (B) Job status gave the task completion rate and failure reporting. (C) Job Counter indicated job progress and additional counter. The progression of the mapper was also displayed graphically at the bottom of web UI page (not shown here). Access to this user interface was through FoxyProxy, described in the Methods.

Throughout the execution of both the BLAST and ortholog estimation steps, we were able to monitor the health of our compute cloud through the user interface for the JobTracker Daemon on the master node (Figure 2). This UI enabled us to see that our map tasks executed properly and to monitor their status as they completed. We were also able to monitor individual running BLAST and ortholog estimation jobs in more detail using the job summary user interface (Figure 3).

Our decision to use High-CPU extra large instances proved both cost and time effective. Although alternatives such as the standard small instances were cheaper per hour, our calculations demonstrated that these alternatives would have required substantially more compute time to complete, ultimately resulting in the same cost totals (Table 3).

**Table 3:** Cost comparison of Amazon's cloud computing instance types.

Instance Type	# Instances	Time * (hours)	Cost ($)
Standard Small (single core)	50	1088	6256 (0.115 per hour per instance)

Standard Large (dual core)	50	544	12512 (0.46 per hour per instance)

Standard Extra Large (4-cores)	50	272	12512 (0.92 per hour per instance)

High-CPU Medium (dual core)	50	544	6256 (0.23 per hour per instance)

High-CPU Extra Large (8 core)	50	136	6256 (0.92 per hour per instance)

Amazon's Elastic Compute Cloud (EC2) can be accessed via a number of differet instance types. For the purposes of our comparative genomics problem, we elected to utilize the extra-large high-CPU instance. Note that the total cost for a small instance is equal to the total of the extra large, despite the large difference in computing time.

## Discussion

Comparative genomics will continue to demand high performance computing solutions. This is especially true as new genome sequencing technologies continue to drive down costs and ramp up volume. The work we present here represents one of the first successful deployments of a standard comparative genomics tool, the reciprocal smallest distance algorithm (RSD), to Amazon's Elastic Compute Cloud (EC2) via the web service Elastic MapReduce (EMR).

To date, most use cases on the cloud have fit the paradigm embodied by the Hadoop and EMR frameworks. The applications are written in Java and are generally "pleasingly parallel" compute problems, such as text or image processing. As such, they conform well to the configuration expected. Our tool, which is likely to be similar to many other comparative genomics algorithms, deviated sharply from these Hadoop and EMR standard use cases. The largest deviation was that the RSD algorithm involves a pipeline of programs written in languages other than Java, including python, perl, and C. At first glance, the streaming functionality provided by EMR appeared to be a viable out-of-the-box solution. However, this function also was not designed to handle complex operations like that inherent to RSD. The original intent of the streaming function was to pass input via standard-in to the mapper for processing, the results of which would be passed via standard-out to the reducer for summation. As such, the object of the mapper was expected to reside within an input directory on the Hadoop Distributed File System used by EMR. Given the complex stream of operations needed by RSD including the need to run a host of programs across whole genomic sequence databases, this straightforward design proved too inflexible. Therefore, we elected to generate, prior to compute cloud configuration, a single input file containing the RSD command-line arguments needed for the set of genomes to be compared. This input file became the object of the mapper, enabling the mapper to read the RSD commands line-by-line and to launch them to compute nodes for processing. This solution provided the flexibility necessary to accommodate a host of programs written in alternative languages aside from Java while retaining the capabilities of the EMR program, most importantly including fault tolerance and job tracking. Because the endpoint of every map task was a file containing the orthologs and evolutionary distances for a specific pair of genomes, a reducer step was not required. However, going forward one could be used for meta-analysis of the results from individual map tasks, such as selecting the most conserved orthologs among a set of genomes, or for assembly and analysis of phylogenetic profiles.

With this solution, we were able to take full advantage of the compute cloud to run RSD in the same way as it would be run on a local Linux compute farm, for a manageable cost. We ran over 300,000 processes in total, computing results for 454 fully sequenced genomes, including 55 new genomes not previously incorporated into the Roundup online genomics resource that employs the RSD algorithm. This successful application demonstrated that the cloud represents an ideal platform for either augmentation of existing local computing hardware, or for complete replacement. We anticipate that other comparative genomics tools that have similar workflows and that are not written entirely in Java will be able to take advantage of the solutions we present here. In particular, the instantiation of the compute cloud, the run configuration steps via the Ruby CLC (Table 1), and our use of the streaming function of EMR should be immediately scalable to other similar problems.

In sum, based on our successful deployment of RSD on Amazon's EC2, we believe that cloud architectures represent an exciting alternative to standard approaches to high performance computing for comparative genomics. It remains to be seen how much of an impact cloud architectures and the "pay-as-you-go" model of computing provided by vendors like Amazon will have on the future of comparative genomics and other fields requiring heavy computation. Our prediction is that the impact will be significant and that within 2 years, a majority of applications like the one studied here will be ported to cloud architectures.

## Conclusions

Cloud computing architectures are rapidly emerging as robust and economical solutions to high performance computing of all kinds. To date, these architectures have had a limited impact on comparative genomics. Here we describe the successful deployment of a commonly used comparative genomics tool, the reciprocal smallest distance algorithm, to the Elastic Compute Cloud (EC2) of Amazon Web Services using the Elastic MapReduce (EMR).

One of the most important components of our deployment was the configuration and use of the streaming function provided by both EMR and Hadoop. By using this function, we were able to capitalize on the technical advantages conferred by EMR/Hadoop, without having to recode our own sequence analysis workflows into Java, and without having to design our own solutions for job queuing, tracking and maintenance. These steps are applicable to virtually any sequences analysis workflow with little or no changes to the configuration settings that we describe. In addition, the procedures we have outlines can be ported to any cloud environment that accommodates standard Linux distributions running Hadoop. Thus, we expect that more and more applications like ours will be running on cloud environments in the near future.

## Methods

### General setup

#### Amazon services requirements

We created an account with Amazon Web Services that provided access to 3 specific products, the Elastic Computing Cloud (EC2) [7], the Elastic MapReduce (EMR) framework [16], and the elastic storage unit (S3) [17]. This account creation process yields "access" and "secret" keys needed to instantiate instances within the EC2 and run the setup and execution procedures detailed below. We used three services provided by Amazon, the EC2, EMR, and S3. The cost per hour for EMR was $0.12, and the cost per hour for use of a single extra large high performance compute instance on EC2 was $0.80. S3 storage cost was $0.15 per GB storage, $0.10 per GB for data transfer in and $0.17 per GB for data transfer out.

#### RSD requirements

Prior to running a comparative analysis between two genomes, it was necessary to download and compile the external programs that are executed within the RSD algorithm, namely blastp, codeml, and clustalW. Once compiled, the executables were placed into a folder called "executables" on a local machine and subsequently compressed into a tarball called "executables.tar.gz". This gzipped tarball was required for later loading to the S3 storage bucket of Amazon's Web Services.

We also downloaded and unpacked to our local machine the complete RSD standalone package from the Roundup website [15]. This cloud-ready version of the reciprocal smallest distance algorithm contains several python programs for both blast- and RSD-specific processes. These programs are listed and described in Table 4. The folder was compressed into a gzipped tarball for later loading to the S3 storage bucket as "rsd_package.tar.gz" and is freely available with the present manuscript as Additional File 1.

**Table 4:** Programs associated with the reciprocal smallest distance algorithm.

Program name	Description
ReadFasta.py	a module used by RSD.py

RSD.py	the main program which executes the RSD reciprocal smallest distance ortholog detection algorithm

BioUtilities.py	a suite of utilities, many of which wrap external programs such as clustalW and PAML

Utility.py	a package used by BioUtilities.py

Blast_compute.py	the main program that builds all-against-all BLAST databases for fast execution of RSD

clustal2phylip	a small perl function that converts clustalw alignment files into files that are recognized by paml

codeml.ctl_cp	the control file required by RSD to properly calculate the maximum likelihood estimates of distance between two protein sequences

execute.py	an error reporter used by RSD

RSD_common.py	the directive file used by RSD

examples	a directory containing examples of inputs and outputs to RSD.py and Blast_co mpute.py.

These programs are required for running the RSD package on a cloud computing platform such as Amazon's Elastic MapReduce. These programs are packaged and available for download at http://roundup.hms.harvard.edu and are also provided as additional files associated with the manuscript.

#### Genome requirements

Genomes were downloaded from NCBI in fastA format, pre-formatted using a program designed to strip out offending characters from the name field, and formatted for blastp using xdformat. The blast indices and pre-formatted fastA files of each genome were placed into a folder named after the organism, e.g. a folder named "Homo_sapiens.aa" was created to hold the human genome fastA file and associated blastp file. All genome folders were then embedded within a parent folder called "Genomes." As in the previous sections this folder was compressed into a tarball for transfer to the S3 storage facility of Amazon Web Services.

#### Moving RSD components to Amazon S3

To transfer files we used the s3 cmd [18]. The s3 cmd tool is an open source command-line tool designed to transfer, download, and manage files within Amazon S3. Once we configured the tool for use on our local cluster, we created a bucket for data uploads/downloads directly on Amazon S3 using the "mb" argument. Then we loaded the gzipped tarballs described in the sections immediately above to this bucket with the "put" argument. Specifically, we ran (1) *s3 cmd mb s3://rsd *and (2) *s3cmdput name_of_tarball s3://rsd/*, where *name_of_tarball *represents one of the three gzipped tarballs described above.

#### Adding log and results folders to S3 bucket

For later processing steps, e.g. results storage and error logging, we created several empty folders within our RSD storage bucket using the s3 cmd: (1) A log folder called "log", (2) a blast_result folder for storing pre-computed blast results required by the RSD algorithm, and (3) an ortholog_results folder for storing the final results of the RSD algorithm.

### The MapReduce algorithm

To handle the computational complexity of RSD, we elected to use the MapReduce algorithm [19]. MapReduce was originally developed at Google for processing on large clusters and was created out of necessity to handle large amounts of raw data to analyze derived data such as summaries of pages crawled per host. The mapreduce algorithm is a two step process that first runs a mapper process designed to distribute jobs to a cluster of a predefined size, and then runs a reducer script to aggregate, store, or otherwise operate on the results generated through the mapper step. We elected to use the EMR web service recently released by Amazon because it adopts the commonly used Hadoop framework [20] and therefore conveys the same advantages, but also provides enhancements to simplify use of the EC2 and interactions with the Amazon S3 storage service. Nevertheless, most of the steps described herein were applicable to both Hadoop and EMR.

### EMR employment of Hadoop deamons

The EMR service wrapped the Hadoop framework, the basis of which was a master-slave mechanism. The EMR framework employed five Hadoop deamons: JobTracker, TaskTracker, NameNode, SecondaryNamenode and DataNode. The Jobtracker and TaskTracker were Java applications running on the master node and slave nodes respectively. The JobTracker node coordinated running processes on the TaskTracker. The NameNode maintained file system name space on the master node, and the DataNode stored the data blocks specific to each slave node. The SecondaryNameNode mirrored the NameNode, acting as a backup in event of a master node failure.

### Configuring the Mappers

Frameworks that implement the MapReduce algorithm, including Hadoop and EMR, originally were designed to run processes written in Java and compiled into jar files. However, both frameworks provide a limited amount of flexibility to run external programs written in other languages via the use of a "streaming" function. Given the complexity of the RSD algorithm and the host of programs needed for its pipeline, none of which were written in Java, we elected to utilize this less mainstream capability of EMR.

When the streaming function is selected, the mapper will operate on a file or files stored directly within the Hadoop Distributed File System (HDFS) and specified by an input option required by the function itself (see Table 1 for options required/accepted by the streaming functions of EMR and Hadoop). Under typical circumstances, these files would be the primary targets for the mapper and reducer tasks. However, the complexity of the RSD algorithm, specifically the number of external programs that needed to be invoked during a comparative analysis of a pair of genomes, did not fit this standard of use. Therefore, we elected to write a program that would generate files containing the precise set of commands needed to run RSD on a specified pair of genomes, exactly as they would be typed on a single unix-based machine. These "runner" files then became the operational targets for the mapper.

**Figure 4:**
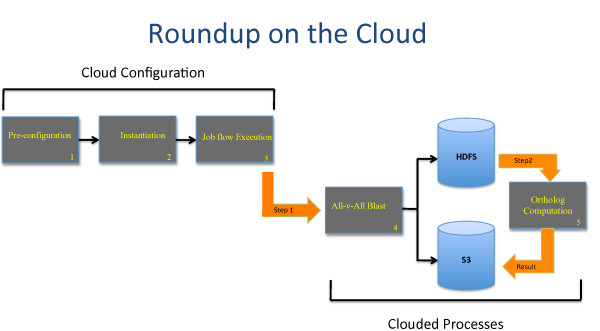
**Workflow for establishment and execution of the reciprocal smallest distance algorithm using the Elastic MapReduce framework on the Amazon Elastic Compute Cloud (EC2)**. (1) Preconfiguration involves the general setup and porting of the RSD program and genomes to the Amazon S3, and configuration of the Mappers for executing the BLAST and RSD runs within the cluster. (2) Instantiation specifies the Amazon EC2 instance type (e.g. small, medium, or large), logging of cloud cluster performance, and preparation of the runner files as described in the Methods. (3) Job Flow Execution launches the processes across the cluster using the command-line arguments indicated in Table 1. This is done for the Blast and RSD steps separately. (4) The All-vs-All BLAST utilizes the BLAST runner and BLAST mapper to generate a complete set of results for all genomes under consideration. (5) The Ortholog computation step utilizes the RSD runner file and RSD mapper to estimate orthologs and evolutionary distances for all genomes under study. This step utilizes the stored BLAST results from step 4 and can be run asynchronously, at any time after the BLAST processes complete. The Amazon S3 storage bucket was used for persistent storage of BLAST and RSD results. The Hadoop Distributed File System (HDFS) was used for local storage of genomes, and genome-specific BLAST results for faster I/O when running the RSD step. Additional details are provided in the Methods.

**Figure 5:**
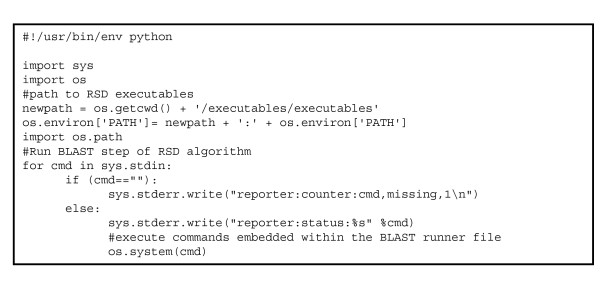
**Example of the mapper program used to run the BLAST and ortholog estimation steps required by the reciprocal smallest distance algorithm (RSD)**. This example assumes a runner file containing precise command line arguments for executing the separate steps of the RSD algorithm. The programs were written in python.

Rather than run BLAST on-the-fly, which is a possibility with RSD, we elected to run BLAST as a separate procedure, compiling a set of precomputed blast results for later use by the evolutionary distance calculations and ortholog identification step of RSD. As such, RSD can be subdivided into two distinct computational steps for the cloud: (1) A BLAST process and, (2) an ortholog estimation process (both steps are depicted in Figure 4). To account for this two-step process, it was necessary to build separate mappers and concomitant runner files. Because of the post- processing steps already embedded within the RSD software, and because the endpoint of each map task was a text file containing the orthologs and associated evolutionary distances generated by RSD, a reducer was not required. An example of a mapper program is provided in Figure 5.

### Configuring the cloud cluster

#### Instantiation

For configuration of the cluster we installed the command-line interface tool called Amazon Elastic Map Reduce Ruby client [21] (Ruby CLC) on a local machine running Ruby vl.8.7. We used Ruby CLC to set several parameters available through EMR for cluster instantiation (Table 1). These same input parameters could also be used for instantiation of a cluster within the Hadoop framework.

To instantiate the cluster, we first ran the EMR program with the following arguments:

--create --alive --name "name of cluster"--num-instances "N" --instance-type "instance type" --log-uri "path to log file" --info '{startupScripts: [{

   name: "s3 location to deamon-memory allocation script",

   args: ["~heap-size-jobtracker = 3072 "]}]}'.

Where the "name of cluster" was any name defined by the user, the num-instances represented the number of instances needed for the run, the instance-type represented the type of instance from the options provide by Amazon Web Services (e.g., small, medium, large, and extra large), and the log-uri argument specified the path to the log folder located in the RSD S3 bucket where error and status messages would be written throughout the run. Finally, the "info" argument allocated memory to the EMR JobTracker Deamon needed to avoid memory related errors with the operation of the compute cloud. We elected to utilize a High-CPU Extra Large Instance 7 GB of memory, 20 EC2 Compute Units (8 virtual cores with 2.5 EC2 Compute Units each), 1690 GB of local instance storage, 64-bit platform.

#### File system setup

EMR in "stream" mode required a folder name be specified for the input option. This could have existed on S3 but in our tests we experienced timeout issues when communicating input from S3, and thus elected to copy the input data directly to the distributed file system used by EMR, the Hadoop Distributed File System (HDFS), using the distcp command. We also elected to use HDFS for temporary storage of pre-computed blast results to enable faster progression from the blast step to the ortholog distance calculation steps of the RSD algorithm (steps 1 & 2 in Figure 4).

#### Monitoring

To monitor the status of the cluster after instantiation, we ran the EMR program with the "--list" and "--active" options specified, which provided a jobflow ID, the status of that job (e.g. "RUNNING"), the exact web address of the master node, and the name of the cluster.

With the web address of the master node, we monitored the status of the cluster through a user interface called FoxyProxy. To access this UI, it was necessary to establish a SOCKS server on the local machine and an SSH tunnel between the local machine and master node. This UI shows the general health of the cluster, including how many jobs were launched, how many are currently running, the number in queue, and more [16]. Examples of user interfaces for the cluster summary and job summary are provided in Figures 2 and 3, respectively.

#### Running

As mentioned above, we elected to split the RSD algorithm into two distinct process flows, one for computation of the BLAST, and the other for estimation of evolutionary distance and determination of orthology. These flows required two separate mapper steps to be run in succession. The results of BLAST step were needed to initiate the ortholog calculation step. However, the ortholog calculation could be run at any later time, as the BLAST results files required by the ortholog step remained available in the S3 storage bucket, providing additional tolerance to cluster failures and avoiding the need to rerun BLAST for the genomes being compared.

The actual cloud computational steps are graphically represented in Figure 4. The commands sent to the EMR program for both step 1 (the BLAST step) and step 2 (the ortholog calculation step) are provided in Table 1.

#### Distributed Cache mechanism for task node configuration

At the time of writing, the EMR framework had a mechanism for copying files and archives to task nodes in time for execution of the job flows. We used this feature to ensure timely delivery of the necessary components of RSD to each slave node in our cluster. To capitalize on the distributed cache mechanism we created an archive of the executables, code, and genomes, as specified in the previous section of this manuscript. In order to distribute the data and code to each task node, we used the following option available via Ruby CLC:

"--cache-archive s3n://rsd_bucket/data. tar.gz#data."

A symlink data folder was then created in the working directory, providing a local copy of genome data and RSD-cloud code on every task node. These files specified were required to be tar archives.

### Portability

While we elected to use EMR for interfacing with the Elastic Compute Cloud, we also confirmed that the all of the above steps could be executed via Hadoop with a few minor changes. Given a machine image containing (1) the base package of Hadoop and (2) the RSD package and genomes of interest, it was first necessary to run the Hadoop start script, start-all.sh to launch the Hadoop Distributed File System and the MapReduce daemons. The command line arguments listed and described in Table 1 were specified in exactly the same way as in EMR, although in Hadoop these parameters are passed to the streaming.jar program, and thus, the --stream option is not required or understood by Hadoop. We also determined that it was beneficial to set the -jobconf mapred.map.tasks.speculative.execution to false. Otherwise, Hadoop would "speculate" that a long running job was malfunctioning and run the same job in parallel, causing performance bottlenecks. Given that Hadoop is open-source and ready for install on standard Linux distributions, the steps described above can be executed on any cloud environment that accommodates Linux.

## Abbreviations

RSD: reciprocal smallest distance algorithm; EC2: elastic compute cloud; EMR: Elastic MapReduce; Ruby CLC: Amazon Elastic MapReduce Ruby client for cluster setup.

## Authors' contributions

DPW conceived, designed and directed the project, conducted the experiments, and wrote the manuscript. PK conducted the experiments and assisted in the writing. VAF, PP, and RP assisted with the cloud experiments and edited the manuscript. PJT helped in project direction and edited the manuscript. All authors read and approved the final manuscript.

## Supplementary Material

Additional file 1 **Cloud-ready reciprocal smallest distance algorithm (RSD)**. complete software stack for RSD-cloud software used to generate the results discussed in the present manuscript. Click here for file
